# Role of *Azolla* in sustainable agriculture and climate resilience: a comprehensive review

**DOI:** 10.3389/fpls.2025.1661720

**Published:** 2025-10-14

**Authors:** Youquan Yang, Yanqiu Yang, Sufang Deng, Zhaoyang Ying

**Affiliations:** Institute of Resources, Environment and Soil Fertilizer, Fujian Academy of Agricultural Sciences, Fuzhou, China

**Keywords:** *Azolla*, sustainable agriculture, biofertilizer, carbon sequestration, methane mitigation, climate resilience, biofuels, bioplastics

## Abstract

Agriculture faces mounting challenges from climate change, soil degradation, and unsustainable agrochemical use, highlighting the need for eco-friendly solutions. *Azolla*, a fast-growing aquatic fern, has emerged as a multifunctional resource for sustainable farming and climate resilience. Through its symbiosis with *Anabaena azollae*, it fixes atmospheric nitrogen, reducing dependence on synthetic fertilizers and improving soil health. *Azolla* also serves as a protein-rich feed for livestock and aquaculture, suppresses weeds and pests in rice systems, and supports water conservation. Beyond agriculture, it contributes to carbon sequestration, mitigates methane emissions, and shows promise in wastewater treatment, bioremediation, and as a feedstock for biofuels and bioplastics. However, large-scale adoption is limited by challenges such as short shelf life, ecological risks, and preservation constraints. This review synthesizes current knowledge on *Azolla*, emphasizing its biological and ecological functions, highlights practical applications across agriculture, livestock, aquaculture, and environmental management, and outlines key research priorities needed to overcome limitations and enable its integration into climate-smart agricultural and environmental systems.

## 1 Introduction

Rapid population growth, climate change, and natural resource depletion create an urgent global challenge for agricultural sustainability (Maja and Ayano, 2021; Ramesh and Rajendran, 2022). Sustainable farming practices aim to balance food production with environmental conservation through strategies that include minimizing chemical use, managing water effectively, and restoring ecosystems while reducing greenhouse gas emissions (Muhie, 2022). Conventional farming practices heavily reliant on synthetic fertilizers and intensive irrigation have led to soil degradation, biodiversity loss, and increased greenhouse gas emissions. The goal is to meet current needs without jeopardizing future generations. Industrial development and agriculture are major contributors to environmental imbalance, necessitating eco-friendly strategies to mitigate climate change impacts. The existing agricultural challenges have escalated the urgency in finding sustainable and regenerative farming methods (Ramesh and Rajendran, 2023; Xu et al., 2024).

Among various alternatives, *Azolla*, a fast-growing aquatic fern, has gained significant attention for its unique biological properties and potential role in sustainable agriculture and climate resilience (Kour et al., 2024). Agricultural systems benefit from *Azolla* integration because it effectively lowers emissions while improving environmental sustainability (Kollah et al., 2016). This aquatic fern forms a symbiotic relationship with *Anabaena azollae* to fix atmospheric nitrogen, enabling it to function as a biofertilizer that reduces synthetic fertilizer use while preventing soil acidification and nitrous oxide emissions (Marzouk et al., 2023). Previous research demonstrates that *Azolla* performs better than inorganic fertilizers (Sood et al., 2012). In addition, *Azolla* contributes to rapid biomass generation, carbon sequestration, and methane reduction (Malyan et al., 2020; Korsa et al., 2024). *Azolla* also purifies water by absorbing heavy metals and pollutants, while serving as a high-protein livestock and aquaculture feedstock (25–33% crude protein), making it both a sustainable and economical supplement. Beyond agriculture, this nutrient-dense resource has uses in industry and healthcare and has even been featured in space diets (Ahluwalia et al., 2002; Prabakaran et al., 2022; Yohana et al., 2023).

The current review highlights *Azolla*’s role in sustainable agriculture and climate resilience by examining its biological properties, nitrogen fixation capacity, carbon sequestration potential, animal feed applications, phytoremediation functions, and industrial uses. The primary focus is on *Azolla*’s role in rice and crop-based systems, while livestock and aquaculture are discussed as complementary but integral components of agricultural systems. In addition, the review identifies key research gaps and proposes future directions to advance *Azolla*-based solutions for climate-smart agriculture.

## 2 Biological and ecological characteristics of *Azolla*

### 2.1 Taxonomy and species diversity

Though its precise classification is still under discussion, Jean-Baptiste Lamarck initially identified the genus *Azolla* in 1783 (Bujak and Bujak, 2024). Initially grouped with Salviniaceae, phylogenetic studies later confirmed its distinct evolutionary lineage (Saunders and Fowler, 1993).

The classification of *Azolla* proves difficult because the genus shows significant morphological variability, vegetative reproduction, and environmental adaptability, which make species identification challenging (Lydia et al., 2023). *Azolla* is divided into two subgenera: *EuAzolla* (*A. filiculoides*, *A. rubra*, *A. microphylla*, *A. mexicana*, *A. caroliniana*) and *Rhizosperma* (*A. pinnata*, *A. nilotica*), differentiated by morphology and reproduction. There are seven extinct and twenty-five fossil species of *Azolla*. The distribution, characteristic features, and uses of different *Azolla* species have been discussed in detail by Kour et al (Kour et al., 2024). Native to America, Africa, Asia, and Australia, *Azolla* has expanded globally due to its invasive nature, though no species are native to Europe. While fossil evidence shows that *Azolla* existed in Europe at one time, it was reintroduced to the continent in 1880 (Korsa et al., 2024; Kour et al., 2024).

Several species have become invasive outside their native ranges, forming dense mats that disrupt ecosystems and economic activities. Examples include *Azolla cristata* (syn. *A. caroliniana*) originated from North and Central America and is now growing in Africa, Asia, and Europe (Korsa et al., 2024; Kour et al., 2024). The native South and Central American *A. microphylla* has been introduced throughout the world (Kour et al., 2024). In contrast, *A. mexicana* remains primarily confined to North and Central America (Kour et al., 2024). *Azolla pinnata*, native to Asia, Africa, and Australia, has been introduced to the USA and South America. *Azolla filiculoides*, tolerant of cold climates, was introduced to China from East Germany in 1977 (Madeira et al., 2019; Kour et al., 2024). Through the introduction, Egypt received *A. caroliniana*, *A. filiculoides*, and *A. pinnata* (Serag et al., 2000b). *Azolla caroliniana* developed into an invasive species in the Danube Delta of Ukraine by 1978 (Prokopuk, 2016). Reflecting evolutionary adaptations, phylogenetic studies utilizing rbcL gene sequences confirm the split of *Azolla* into *Euazolla* and *Rhizosperma* (Mahmood et al., 2020). Species like *A. pinnata* and *A. filiculoides* are widely used in agriculture, while others remain underexplored for potential applications (Kour et al., 2024).

### 2.2 Growth and reproduction

The aquatic fern *Azolla* doubles its biomass roughly every 2 to 5 days, producing 3–9 tons of dry matter per hectare annually (Lumpkin and Plucknett, 1980; Wagner, 1997). Critical factors affecting *Azolla* growth and nutrient composition have been discussed in detail previously (Marzouk et al., 2023). Briefly, growth depends on temperature, light, nutrients, and water pH, with an optimum of 18–28°C; growth slows below 15°C and stops above 35°C (Sadeghi et al., 2013). Its symbiosis with Anabaena *azollae* enables survival in low-nitrogen conditions, though it thrives in nutrient-rich waters (Lechno-Yossef and Nierzwicki-Bauer, 2002).

**Figure 1:**
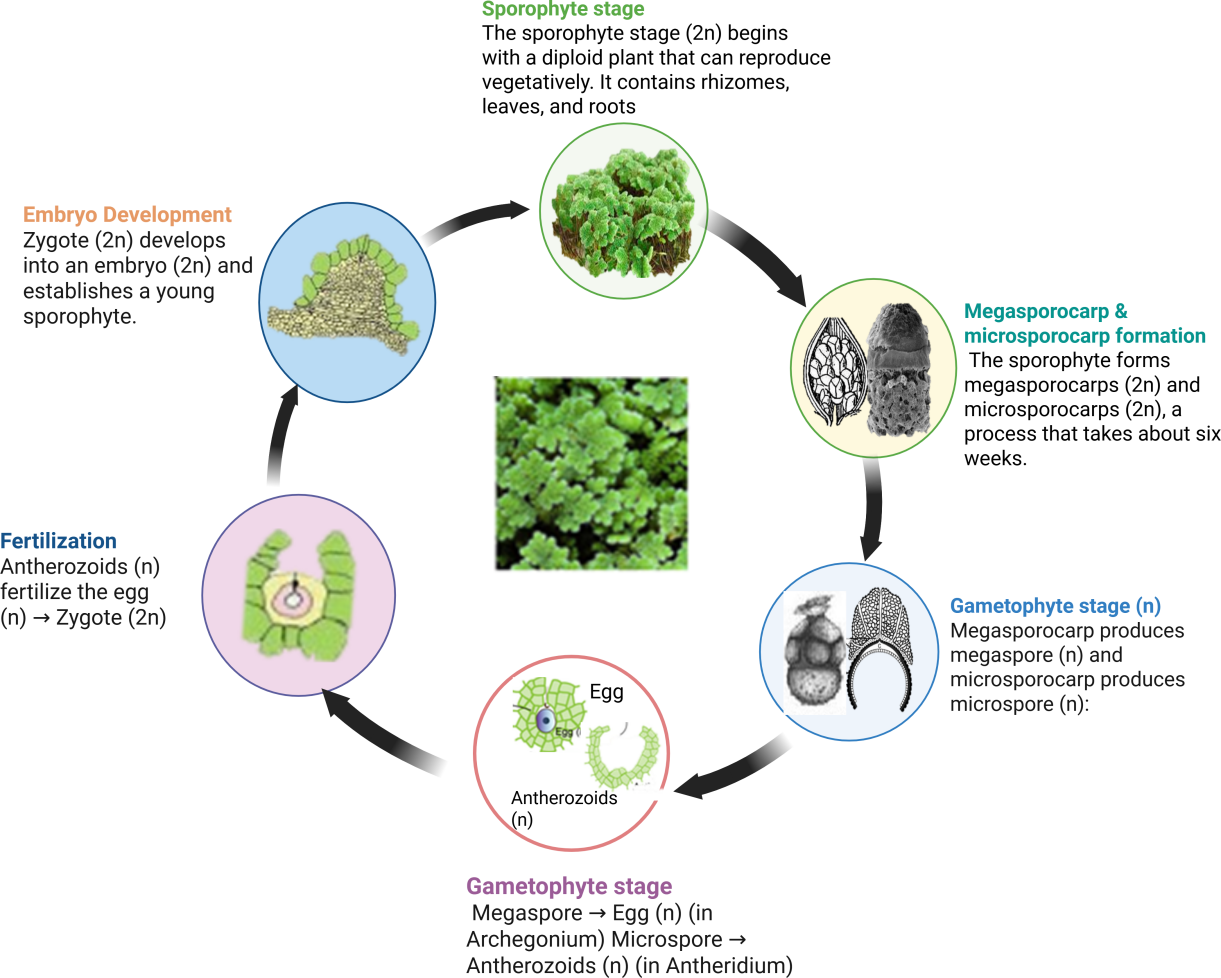
An example of the general life cycle of *Azolla* species showing various developmental stages. The sporophyte had a rhizome, leaves, and roots. Rhizome develops dense leaves containing cyanophycean algae on the upper surface. Adapted from (Sebastian et al., 2021), with permission from John Wiley & Sons.

Reproduction occurs mainly through vegetative propagation, via detachment of rhizome branches, which allows rapid spread. Sexual reproduction is less common, involving heterosporous sporocarps containing microspores and megaspores (Sebastian et al., 2021; Schluepmann et al., 2022). The life cycle of *Azolla* varies by species. The process of sexual reproduction starts when paired sporocarps develop from shoot apical meristems, including both a megasporocarp with one megasporangium and a microsporocarp with several microsporangia (Dijkhuizen et al., 2021; Schluepmann et al., 2022). During sporocarp formation, *A. azollae* is recruited into the indusium cap near root-forming branches. While microsporocarps discharge massulae, including microspores, megasporocarps develop into megagametophytes, creating archegonia (**Figure 1**). Fertilization occurs when flagellate gametes reach the archegonia, leading to diploid growth, though the timing of microgametophyte and gamete development remains unclear (Schluepmann et al., 2022).

Sporocarps in *A. filiculoides* can remain viable for up to four years at 4°C or indefinitely if dried and cryopreserved at -80°C, whereas fragile, water-rich sporophytes cannot be stored (Li et al., 2018). The shift to the haploid phase happens during the start of sporangial development, which depends on light conditions, temperature, and nutrient levels (White, 1971). Unlike seed plants, *Azolla* exhibits high plasticity in sporangial meristem formation, occurring in both sporophytes and gametophytes. Different *Azolla* species demonstrate variable sporangial responses when exposed to distinct environmental stimuli. For example, *A. filiculoides* produces sporocarps when exposed to far-red light, but this formation stops under open-field red light conditions (Dijkhuizen et al., 2021). Sporocarp formation is likely controlled by a conserved phase transition network involving regulatory elements known from seed plants, such as MIKCC, AP2, and GAMYB-microRNA319 interactions (Ambrose and Vasco, 2016). The processes controlling spore germination and gametophyte growth are probably controlled by the sporocarp itself, given the protected nature of *Azolla* gametophytes. During periods of environmental stress, sporocarps descend to the depths of aquatic environments and stay dormant until the conditions improve (Sood and Ahluwalia, 2009). Different *Azolla* species thrive in diverse habitats. *Azolla pinnata*, for instance, likes higher temperatures; *A. filiculoides* may survive in colder temperatures (Metzgar et al., 2007). However, other factors, such as high salinity, UV radiation, and heavy metals, can affect their growth (Korsa et al., 2024). *Azolla* plants in cold regions submerge during the winter and then emerge when the temperature increases. It can change their color from grey green to red-purple when exposed to intense sunlight. *Azolla* thrives in freshwater bodies like ditches, swamps, lakes, and rivers and is also called duckweed, mosquito, or water fern (Kour et al., 2024). While modern species are free-floating, fossils suggest that extinct species had suberect growth (Watanabe and Berja, 1983). Molecular research highlights genetic traits that enhance stress resistance, offering potential for selective breeding. Its sporophyte phase features a floating rhizome with leaf-like fronds and submerged roots.

### 2.3 Symbiotic nitrogen fixation

**Figure 2:**
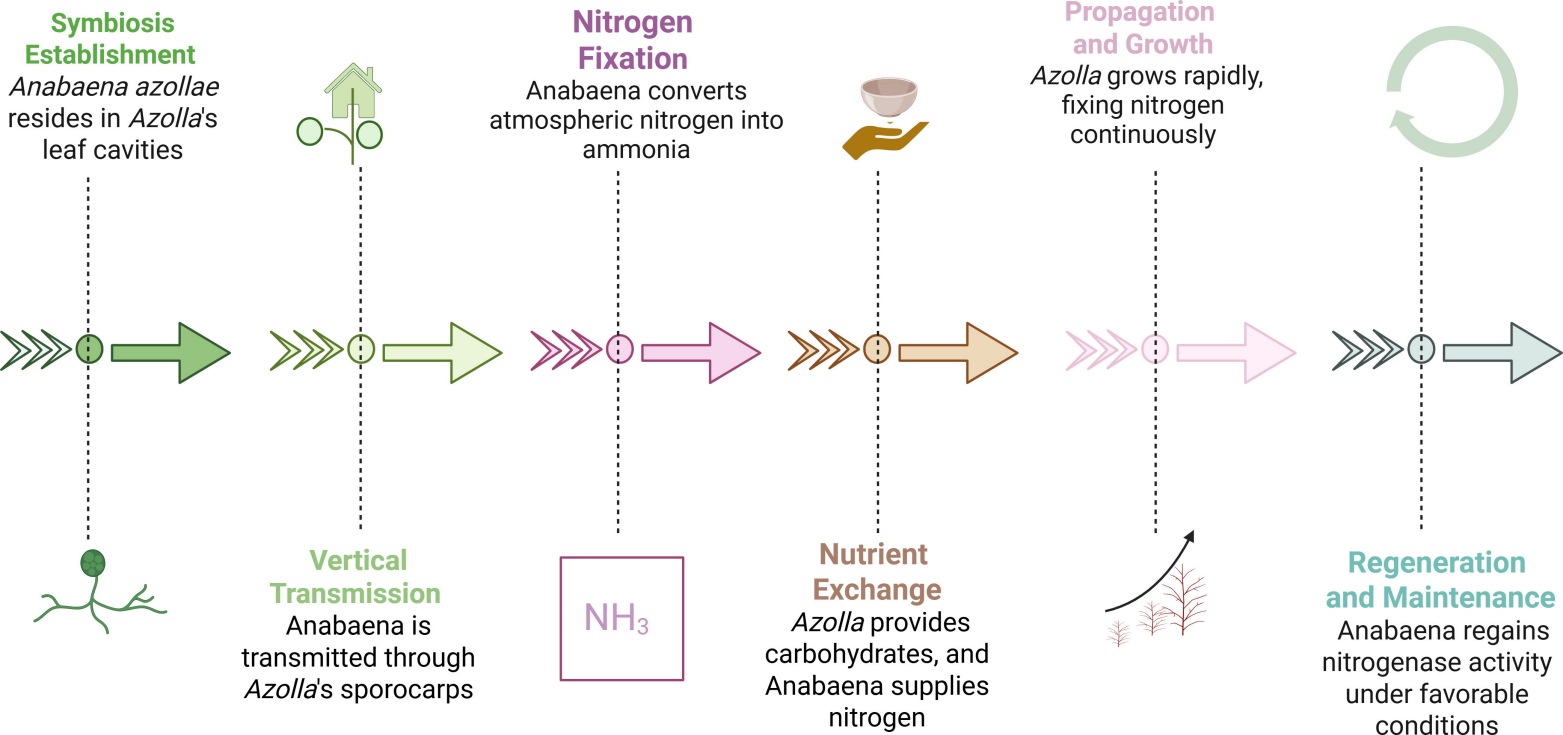
Example of *Azolla*-*Anabaena* symbiosis process (Peters and Meeks, 1989).

The nitrogen-fixing cyanobacterium *A. azollae* resides in specialized cavities of *Azolla* leaves, forming a mutualistic symbiosis first observed by Strasburger in 1873 and later described by De Bary (Carrapiço, 2010; Kour et al., 2024). This relationship enables *Azolla* to thrive in nitrogen-poor waters and function as an effective organic fertilizer (**Figure 2**) (Peters and Meeks, 1989).

Molecular studies confirm the long-term coevolution of *Azolla* and its cyanobiont (Qiu and Yu, 2003; Papaefthimiou et al., 2008; Pereira and Vasconcelos, 2014). Phylogenetic analyses indicate a single evolutionary origin of the symbiosis, which has remained stable for over 100 million years (Bujak and Bujak, 2024). Vertical transmission through megasporocarps ensures that each new generation inherits its cyanobiont without external inoculation, maintaining high nitrogen-fixation efficiency (Ran et al., 2010).

The system functions without requiring external inoculation while preserving strong nitrogen-fixing efficiency (Carrapiço, 2010). The propagation of *A. azollae* within *Azolla* ferns depends on its apical colony in the shoot apex and the movement of its motile filaments (hormogonia) to organ initials like leaf cavities and sporocarps. The regulation of hormogonia movement and cell differentiation in *Azolla* is mostly unknown. Some evidence suggests that secretory trichomes and deoxyanthocyanins might affect this process (Cohen et al., 2002). The leaf cavity functions as a microhabitat that controls oxygen levels to protect nitrogenase from deactivation, thereby enabling nitrogen fixation. *Azolla*’s leaf cavities and sporocarps host a diverse microbial (Rai et al., 2002). Some studies suggested that some bacteria synthesize plant hormones like indole-3-acetic acid, which can improve the growth of *Azolla* (Kumar et al., 2022). Therefore, the *Azolla-Anabaena* relationship forms a complex microbial network that functions as a superorganism beyond its initial binary symbiosis (Carrapiço, 2017). *Azolla* maintains association with one cyanobacterial species, which contrasts with legumes hosting multiple symbiotic partners and prompts further investigation into its coevolution and metabolic interactions. Genetic research has identified regulatory differences in nitrogen fixation, which may lead to agricultural improvements (Pabby et al., 2003; Devaprakash et al., 2024).

## 3 Role of *Azolla* in sustainable agriculture

*Azolla* has long been used in agriculture mostly for water conservation, weed control, and soil fertility enhancement. Its use as a biofertilizer in rice systems dates back to China’s Tang Dynasty (618–907 AD), when farmers applied it as green manure to boost rice yields (Lumpkin and Plucknett, 1980). By the Ming Dynasty (17th century), its use had become widespread (Tarif, 2021; Kour et al., 2024). Cultivation began in Fujian and Guangdong, later spreading south of the Yangtze; after the establishment of the People’s Republic, its use expanded northward as both manure and animal feed. In central and southern China, it is still grown before early rice planting. In Vietnam, the use of *A. pinnata* as green manure dates back to the 11th century, predating its spread to China, India, and the Philippines (Tarif, 2021). Oral traditions suggest its domestication in La Van village, Thai Binh province, where villagers reared *Azolla* starter cultures from April to November and sold them to farmers at premium prices before the Vietnamese revolution (Watanabe, 1982; Tarif, 2021).

**Figure 3:**
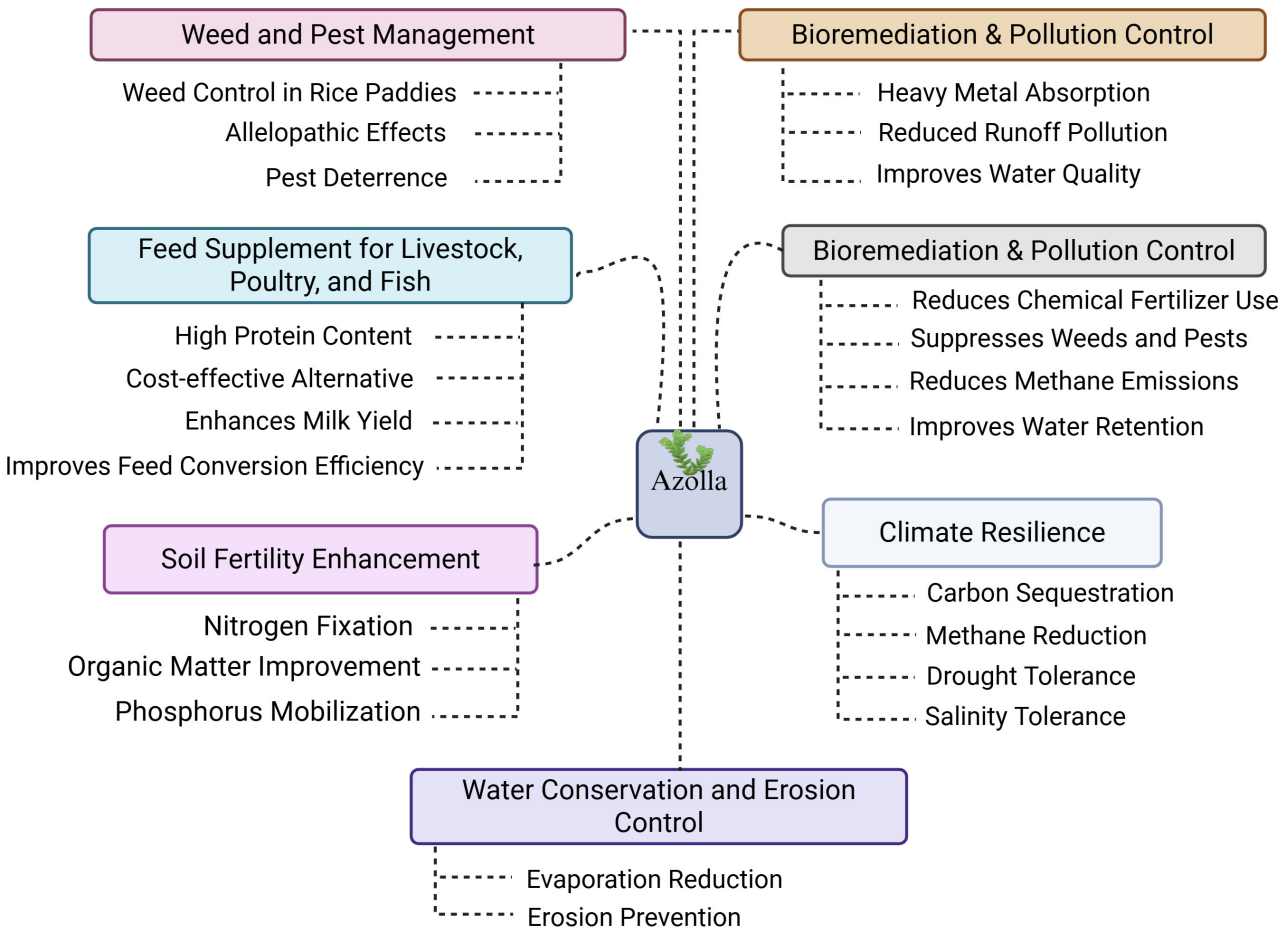
Examples of *Azolla*’s multifunctional benefits in agriculture and environmental sustainability.

The symbiotic relationship between *Azolla* and *A. azollae* enables *Azolla* to function as a natural source of nitrogen through direct atmospheric nitrogen fixation into the plant. In flooded rice systems, fixation rates of 2–4 kg N per hectare per day have been reported, substantially reducing the need for synthetic fertilizers and positioning *Azolla* as an important component of sustainable agriculture (Pabby et al., 2003). This biologically sourced nitrogen not only lowers production costs but also minimizes environmental contamination compared to chemical fertilizers (Wagner, 1997). In rice paddies, the dense floating mat of *Azolla* suppresses weeds by blocking sunlight and reduces water loss through evaporation, thereby decreasing reliance on herbicides and manual weeding (Pabby et al., 2003). Its water-retention capacity also helps maintain soil moisture in drought-prone areas (Peters and Meeks, 1989). Beyond soil fertility, *Azolla* has long been used as livestock and aquaculture feed due to its protein-rich composition and balanced amino acid profile. More recently, it has been adopted in Iran, Africa, and parts of Europe for rice cultivation and aquatic farming (Madeira et al., 2016). Research in the 20th century further revealed its potential in carbon sequestration, organic farming, and phytoremediation. Its ability to absorb heavy metals and pollutants highlights its value in environmental remediation (**Figure 3**), reinforcing its role in modern sustainable agricultural systems (Yao et al., 2018).

### 3.1 Sustainable biofertilizer for soil health

Through its symbiosis with *A. azollae*, *Azolla* contributes 30–60 kg N ha^-^¹ per season via biological nitrogen fixation, improving soil fertility and raising nitrogen-use efficiency by up to 70% compared with synthetic fertilizers (Kollah et al., 2016; Kour et al., 2024) (**Table 1**). With a rapid growth rate that doubles biomass in 3–5 days, *Azolla* fixes 1.1–3.5 kg N ha^-^¹ day^-^¹, exceeding many legumes (Pillai, 2001; Vijayan et al., 2024). When used as a dual crop in rice paddies, it supplies 40–60 kg N ha^-^¹ per cycle (Adhikari et al., 2020). *Azolla* inoculation with 16.5–17.5 t fresh weight ha^-^¹ fixes 52.5–55.1 kg N ha^-^¹, while 12.2 t dry matter ha^-^¹ provides 33.8 kg N ha^-^¹ (Raja et al., 2012). The efficiency of nitrogen fixation varies depending on climatic conditions, floodwater nutrient levels, *Azolla* species, and rice growth stages (Kour et al., 2024).

**Table 1:** Examples of some studies that report the use of *Azolla* for soil improvement.

Application rate	Application method	Observations	References
10–90 g/kg soil	Incorporated into soil, incubated at 25°C for 60 days	Increased soil pH, organic matter, and nutrient availability (N, P, K, Ca, Mg)	(Bhuvaneshwari and Kumar, 2013)
1 t/ha	Applied to rice fields 7–10 days after transplanting	Increased N fixation (up to 600 kg N/ha), improved water retention, porosity, and cation exchange	(Nayak et al., 2004)
300 kg/ha	Incorporated into rice fields	Enhanced nitrogen availability in soil	(Kandel et al., 2020)
5 t/ha (dry matter)	Applied as compost with/without synthetic fertilizers	40% NPK+60% *Azolla* compost improved yield, nutrient uptake, and growth	(Seleiman et al., 2022)
12.5 kg fresh *A. imbricata* per tree	Incorporated into mandarin orange garden soil at 10 cm depth	Increased soil pH, organic carbon, available nitrogen, phosphorus, NH_4_^+^-N, and NO_3_^-^-N; enhanced nitrogen functional bacterial diversity	(Lu et al., 2017)
10 t/ha	K-enriched *Azolla* incorporated into soil (60% & 100% moisture)	Increased organic carbon, N, P, K; better results at 60% moisture	(Muruganayaki et al., 2019)
500 kg/ha	Used as dual crop in rice fields	Increased soil nitrogen by 50 kg/ha, reducing nitrogen fertilizer needs by 20–30 kg N/ha	(Verma et al., 2022)
Not specified	Used as green manure with rice	Improved N, P, K, organic C, and microbial activity	(Marzouk et al., 2023)
20 t/ha	Incorporated before rice transplanting	Enhanced organic C, N, P, cation exchange, porosity, and water retention	(Awodun, 2008)
3 t/ha (fresh weight)	Combined with 300 kg urea-N/ha	Increased nitrogen recovery by 59%, reduced NH_3_ loss by 12%, and enhanced rice yield by 14%	(Yao et al., 2018)
10, 20, 30 t/ha	Applied with phosphate-solubilizing bacteria	Increased available P, plant P uptake, and productive rice tillers	(Pujawati et al., 2023)
Not specified	Intercropping of *Azolla* and rice	Increased the organic carbon, available phosphorus and total nitrogen of soil	(Singh and Singh, 1990)
6% *Azolla* extract+20 t/ha biochar	Biochar was incorporated; *Azolla* was foliar-sprayed.	Improvement in soil organic matter, water retention, CEC, microbial biomass	(Al-Sayed et al., 2022)
Not specified	Used as green manure in rice fields	Improved organic matter, N, and fertility, leading to higher yields	(Singh and Singh, 1987)
NP+½ K through *Azolla* GM+½ K through mulching	*Azolla* incorporated as green manure+mulched application	Increased water-soluble K, available K, and exchangeable K	(Jha et al., 2023)

Beyond nitrogen, after incorporation, *Azolla* enhances soil organic matter, microbial activity, and physical structure. Its decomposition increases aggregate stability, porosity, water retention, and permeability while reducing bulk density (Marzouk et al., 2023; Sun et al., 2024; Ansabayeva et al., 2025). These changes support higher crop yields. Humus derived from *Azolla* improves cation exchange capacity and nutrient availability (Ca²^+^, Mg²^+^, K^+^, P) (Kour et al., 2024). The breakdown of *Azolla* in soil helps various nitrogen-fixing bacteria and fungi to flourish, which in turn enhances nutrient cycling and crop nutrition (Samarajeewa et al., 2005; Adhikari et al., 2020).

Compared to synthetic nitrogen sources, *Azolla*-derived nitrogen is more efficient in terms of plant uptake and fertilizer use efficiency (Seleiman et al., 2022; Marzouk et al., 2023). Integrating organic and inorganic fertilizers sustains crop productivity and enhances soil health (Pushpanathan et al., 2004). Several studies demonstrate that mixing *Azolla* into soil helps improve nitrogen mineralization and its usage. The efficiency of fertilizers is enhanced when *Azolla* is added to the soil (Bhuvaneshwari and Singh, 2015; Adhikari et al., 2020). A previous study reported that the application of 86 kg N ha^-^¹+1000 kg *Azolla* ha^-^¹ increased rice growth by 15.54%, yield by 25.49%, and nitrogen-use efficiency (Safriyani et al., 2020).

Beyond nitrogen, *Azolla* increases phosphorous availability by 20–30%, hence very helpful for soils lacking phosphorus (Raja et al., 2012). With 3–5% nitrogen and 3–6% potassium in its biomass, it exceeds traditional green manures in nutrient value (Kour et al., 2024). *Azolla* breakdown increases urease and phosphatase activity, encouraging mineralization of nutrients (Herath et al., 2023). Moreover, *Azolla* is essential for the control of soil pH since it reduces acidification in acidic soils and increases phosphorus solubility in alkaline soils, thus boosting the availability of nutrients in several agroecosystems (Herath et al., 2023; Marzouk et al., 2024).

*Azolla* reduces runoff, prevents erosion, and improves aggregation, particularly when cultivated along contour lines or irrigation channels (Adhikari et al., 2020; Herath et al., 2023). Floating mats in rice paddies protect against sediment loss, while fine rootlets deposit silt in wetlands and channels, limiting nutrient depletion (Raja et al., 2012; Kollah et al., 2016). These processes enhance root development and water-use efficiency in rice fields (Razavipour et al., 2018).

*Azolla* contributes to abiotic stress management. It tolerates moderate salinity, removing excess salts from soil and water (Sadeghi et al., 2014). Its mats limit evaporation and salt buildup, reducing crop salinity stress (Serag et al., 2000a). Compost from *Azolla* enhances rice growth on saline soils by releasing organic acids that improve nutrient availability while aiding salt removal (Razavipour et al., 2018). Collectively, these properties establish *Azolla* as a cost-effective alternative to conventional soil amendments.

#### 3.1.1 *Azolla* application in rice cultivation

*Azolla* significantly enhances rice grain yield, straw yield, caryopsis formation, and dry matter production when incorporated into paddy fields (Pabby et al., 2003). It is applied either as green manure before transplanting or as a dual crop after transplanting, with the latter being more widely adopted due to its greater agronomic benefits (Kimani et al., 2022). In the green manure system, *Azolla* is collected from nurseries, ponds, or ditches and applied 2–3 weeks before rice transplanting. Healthy, fresh *Azolla* inoculum is essential for efficient production, with inoculum density playing a crucial role (Adhikari et al., 2020). Singh recommends 2 t ha^-^¹, while in Vietnam, 5 t ha^-^¹ or more is preferred (Singh, 1981; Marzouk et al., 2023). Insufficient density can lead to overgrowth by algae and weeds. Various *Azolla* cultivation methods are used globally, with Vietnam favoring the half-saturation method. *Azolla pinnata* reaches a saturated density of 10–20 t ha^-^¹. The process begins by spreading inoculum at 0.5 kg m^-^². After one week, when the surface is fully covered, half of the *Azolla* is transferred to a new area of equal size. Within another week, both areas will reach full coverage. This cycle is repeated, doubling the covered area each time, leading to exponential expansion (Watanabe, 1982).

*Azolla* forms a thick mat that decomposes into the soil, supplying 20–40 kg N/ha and enhancing soil fertility and crop yields (Kulasooriya and De Silva, 1977; Watanabe et al., 1977). In dual cropping systems, introducing 0.5–1 t/ha of fresh *Azolla* after transplanting allows a dense mat to form within 15–20 days. Decomposing in 8–10 days, it releases nitrogen to support rice growth throughout the crop cycle, providing approximately 30 kg N/ha per cycle. To optimize nitrogen fixation, superphosphate (20 kg/ha) is applied in split doses (Watanabe et al., 1977; Yadav et al., 2014).

Yield impacts are well-documented. *Azolla* compost at 5% soil weight raised grain yield by 13.8% (Razavipour et al., 2018). A 1975 review of 1,500 trials in southern China reported yield increases of 600–750 kg ha^-^¹ (FAO-Rome, 1979; Liu, 1979). In Chekiang Province, 90% of 422 trials reported an average yield gain of 700 kg ha^-^¹ (18.6%) (Liu, 1979). Vietnamese studies found 1 t fresh *Azolla* increased yield by 28 kg, with 20 t ha^-^¹ raising yields by 0.5 t ha^-^¹ (Ventura et al., 1992; Nyoni, 2011). Dual cropping improved yields by 36–38% (Barthakur and Talukdar, 1983), while *A. pinnata* specifically increased grain yield by 6–29% (Moore, 1969). Integrating *Azolla* with neem cake-coated urea further maximized yield (Sukumar et al., 1988). Several other studies have demonstrated substantial yield improvements associated with *Azolla* application. Peters found that using *Azolla* as a monocrop biofertilizer increased rice yield by 112% compared to unfertilized controls, while intercropping with rice resulted in a 23% yield increase (Peters, 1978). When applied as both a monocrop and an intercrop, the yield increase reached 216%. Singh observed that the application of 30–40 kg N/ha from ammonium sulphate or 8–10 t/ha of fresh *Azolla* led to a 47% increase in grain yield (Singh, 1977). A review of multiple studies indicated that *Azolla*-based cropping systems increased grain yields by 14–40%, while monocropping during the fallow season resulted in a 15–20% yield increase (Samal et al., 2020).

Studies also indicate that incorporating *Azolla* enhances nitrogen recovery by 49–64% while reducing nitrogen loss by 26–48% (Yao et al., 2018). The nitrogen fixation capacity of *Azolla* varies across species, with *A. filiculoides* fixing 128 kg N/ha in 50 days, *A. pinnata* fixing 0.3–0.6 kg N/ha/day, and *A. africana* fixing 0.6–1.8 kg N/ha/day (Kumarasinghe and Eskew, 1993). Basal applications of 10–12 t ha^-^¹ increased soil N by 50–60 kg ha^-^¹, reducing fertilizer needs by 30–35 kg ha^-^¹ (Roy et al., 2016). Similarly, adding 500 kg/ha of green *Azolla* has been reported to raise soil nitrogen by 50 kg/ha, further reducing the need for nitrogenous fertilizers by 20–30 kg/ha (Roy et al., 2016). Additionally, *Azolla* application reduces NH_3_ volatilization by 12–42%, minimizing nitrogen loss in flooded rice systems (Yao et al., 2018).

*Azolla*’s effectiveness in rice production extends to its role in nitrogen management strategies. Studies indicate that applying *Azolla* with reduced nitrogen levels achieves yields comparable to full nitrogen applications, making it a viable alternative to synthetic fertilizers. For instance, applying 60 kg N/ha from *Azolla* along with 30 kg N/ha from urea resulted in yields equivalent to those obtained with a full 60 kg N/ha urea application (Setiawati et al., 2020). Additionally, *Azolla* lowers flooded water pH and temperature, contributing to reduced NH_3_ volatilization and improved nitrogen use efficiency (Yao et al., 2018). Beyond its contribution to nitrogen supply, as discussed in section 3.1, *Azolla* improves soil structure, enhances organic matter accumulation, and increases the availability of essential micronutrients such as Zn, Fe, and Mn (Subedi and Shrestha, 2015). Moreover, it releases plant growth regulators and vitamins that further promote rice growth and yield (Thapa and Poudel, 2021).

Integrated systems further boost sustainability*. Azolla*, integrated with rice, fish, and ducks, enhances nutrient cycling, soil fertility, and pest control while reducing chemical inputs. This sustainable system improves productivity and biodiversity while minimizing environmental impact (Sanginga and Van Hove, 1989; Van Hove, 1989). In the rice-fish-*Azolla* system, *Azolla* acts as a biofertilizer and fish feed, improving rice and fish production (Shanmugasundaram and Ravi, 1992). *Azolla* application at 2 t/ha increased yields and the benefit-cost ratio (1.88) (Sivakumar and Solaimalai, 2003). Fish stocked at 6,000/ha with *Azolla* feed generated a net income of $258/ha, surpassing rice monoculture by $51 (Van Hove, 1989; Cagauan and Pullin, 1994).

**Figure 4:**
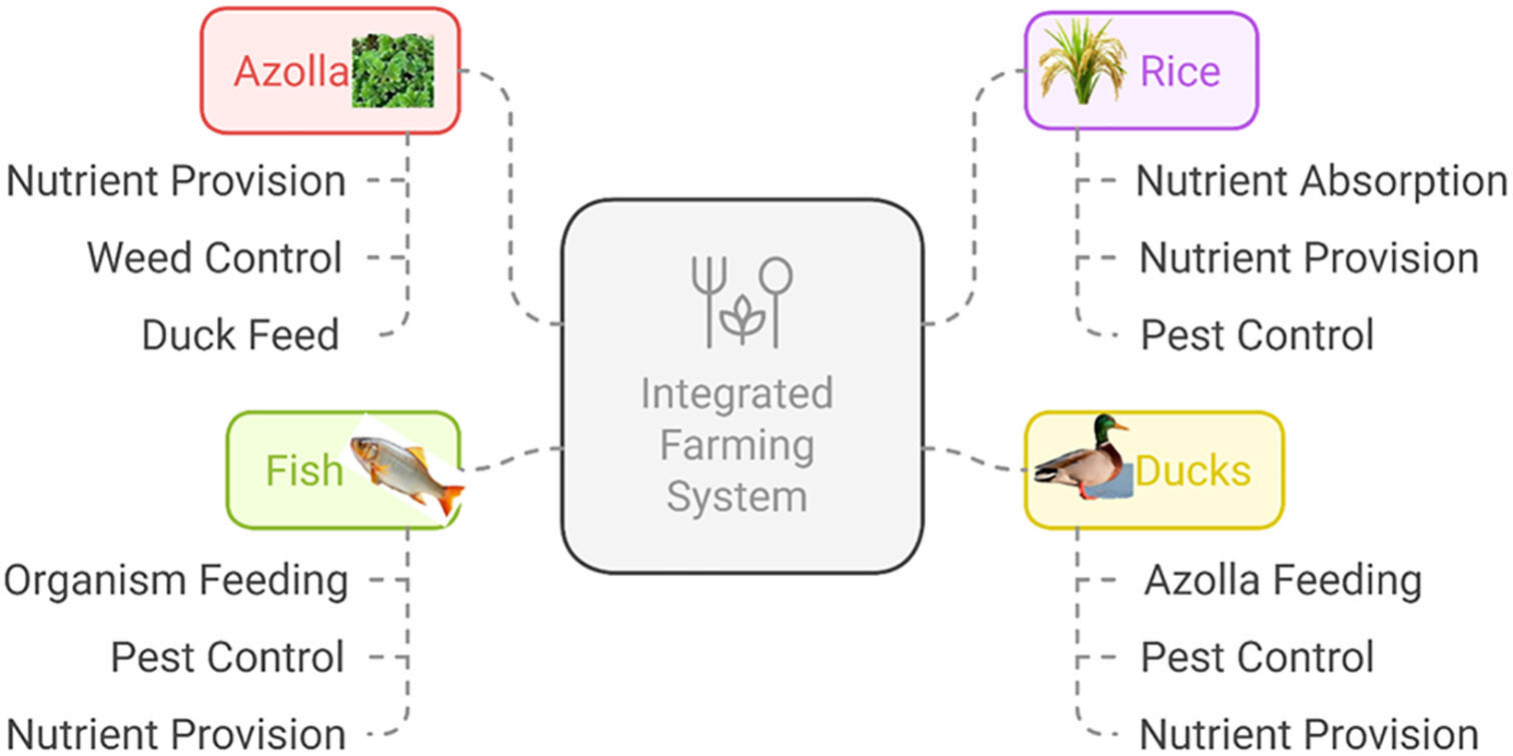
Schematic representation of rice–*Azolla*–duck–fish Interrelationships in an integrated farming system.

The rice-fish-*Azolla*-duck system (**Figure 4**) builds upon this approach by introducing ducks, which help control weeds and pests while enriching soil with their droppings (Sow and Ranjan, 2020). Ducks introduced 15–20 days after rice transplantation reduce reliance on pesticides, while *Azolla* supports soil health and serves as feed for both fish and ducks (Lumpkin and Plucknett, 1980). Fish benefit from organic matter derived from duck manure and decomposed *Azolla*, improving growth and productivity (Sow and Ranjan, 2020). Studies report up to a 58% increase in rice yield compared to monoculture due to improved nutrient cycling and pest control (Cagauan et al., 2000).

These systems significantly enhance pest control efficiency, reducing populations of rice pests such as green leafhoppers, brown planthoppers, stem borers, leaf folders, whorl maggots, and gall midges (Cagauan et al., 2000; Sapcota and Begum, 2022). Combined use of *Azolla*, fish, ducks, liquid biofertilizer, and nano-urea extends nutrient availability and boosts physiological traits, delivering high productivity with reduced chemical inputs (Sow and Ranjan, 2020).

Finally, *Azolla* benefits extend to non-rice crops. In taro (*Colocasia esculenta*), its use as green manure significantly raised yields (Tekle-Haimanot and Doku, 1995). In rice–wheat systems, it enhanced wheat yields, particularly when combined with Sesbania (Mahapatra and Sharma, 1989). It is also harvested from water bodies for use in wheat and vegetables (Pabby et al., 2003). In banana plantations, it serves as nutrient rich-mulch (Van Hove, 1989; Wijeysingha and Amarasinghe, 2023).

### 3.2 *Azolla* in water conservation, weed, and pest control

*Azolla* forms a dense floating mat on the water surface, reducing evaporation by up to 60% and conserving soil moisture (Kour et al., 2024). By covering the water surface, *Azolla* limits sunlight penetration, thereby lowering water temperature and evaporation rates, which is particularly beneficial in regions with water scarcity or irregular rainfall (Marzouk et al., 2023). Additionally, In non-flooded cropping systems, *Azolla* can also be applied as a living mulch, improving soil water retention and reducing moisture loss (Raja et al., 2012).

The thick *Azolla* mat prevents sunlight from reaching submerged weed seeds, inhibiting germination and growth. This eco-friendly method provides an alternative to herbicides (Adhikari et al., 2020). Previous studies show that *Azolla* can reduce weed biomass by up to 50% (Herath et al., 2023). Since weeds compete with rice for nutrients, light, and water, infestations can cause yield losses of 16–100% depending on severity (Geetha et al., 2022). Unlike herbicides such as 2,4-D, glyphosate, and propanil, which harm non-target organisms and degrade soil and water quality (Shekhawat et al., 2022), *Azolla* offers a safer alternative. Its potential in smallholder systems, first recognized in 1927, remains underexploited (Herath et al., 2023).

*Azolla* effectively suppresses multiple weeds, including *Echinochloa crus-galli, Cyperus serotinus, Monochoria vaginalis, Eclipta prostrata, Fimbristylis miliacea, and Cyperus rotundus* (Herath et al., 2023). By blocking light, altering microclimate (reducing evaporation and soil temperature), and improving soil structure when incorporated as green manure, *Azolla* reduces weed competition and enhances rice growth (Marzouk et al., 2023). *Azolla* also exhibits allelopathic properties that inhibit weed germination and growth. It releases secondary metabolites such as phenolic compounds, flavonoids, and tannins, which suppress invasive weed species (Bahadur et al., 2015; Ameena et al., 2024). Some studies have demonstrated that *Azolla* extracts negatively affect root elongation in weeds, further reinforcing its potential as a natural weed management tool (Herath et al., 2023).

The floating *Azolla* mat also disrupts pest life cycles. It prevents mosquitoes from laying eggs on water surfaces, lowering mosquito populations and reducing vector-borne disease risk (Kour et al., 2024). It interferes with the breeding of rice stem borers and leafhoppers, which require open water for egg-laying (Marzouk et al., 2023). Furthermore, *Azolla* supports beneficial organisms such as predatory insects, frogs, and fish, which feed on pest larvae, thereby enhancing natural pest control mechanisms and reducing the need for chemical pesticides (Raja et al., 2012). In integrated rice–fish systems, *Azolla* not only acts as biofertilizer and fish feed but also suppresses weeds and pests, reducing chemical inputs and maintaining ecological balance (Pabby et al., 2003). *Azolla* extracts possess antifungal and antibacterial properties, helping to mitigate plant diseases such as rice blast and sheath blight (Phukon et al., 2017). *Azolla* bioactive compounds have also demonstrated effectiveness against fungal infections in other crops, highlighting its potential in sustainable disease management (Pereira et al., 2015).

### 3.3 *Azolla* as animal feed

*Azolla* is a nutrient-rich, high-protein feed supplement for livestock, poultry, and fish. It provides an excellent amino acid profile, high digestibility, and essential micronutrients, serving as a sustainable alternative to conventional protein feeds such as soybean meal and fish meal, which are costly and environmentally intensive (Kour et al., 2024).

The nutrient composition of *Azolla* varies by species, geography, production methods, and soil conditions (Shaltout et al., 2012; Bhavyasree, 2015). Several studies have reported different compositions of different species of *Azolla* (Katole et al., 2017; Khursheed et al., 2019). *Azolla microphylla* and *A. filiculoides*, which are most commonly used, contain 91.77%–92.25% moisture, 3.9%–5.2% crude protein, 0.6%–1.8% crude fat, and 2% ash (Bhaskaran and Kannapan, 2015). On a dry matter basis, *Azolla* has 25–35% crude protein, 10–15% minerals, and up to 10% amino acids, making it comparable to commercial protein feeds (Marzouk et al., 2023). Despite its high nutritional value, inclusion in animal diets is generally limited to 25% due to anti-nutritional compounds.

Among species, *A. pinnata*, native to warm regions, has higher polyphenolic tannins, reducing digestibility, while *A. filiculoides*, found in the Americas and Europe, contains lower polyphenols and higher protein, making it a better protein source (Brouwer et al., 2018)). *Azolla* is also rich in lysine, methionine, and arginine, essential for muscle growth, as well as key minerals like calcium, phosphorus, magnesium, iron, and potassium, which support bone health and metabolism (Herath et al., 2023; Yohana et al., 2023). High beta-carotene and vitamin A levels enhance vision, immunity, and reproduction (Adhikari et al., 2020). Its low lignin content (<5%) ensures high digestibility for both ruminants and non-ruminants (Raja et al., 2012), while bioactive compounds such as flavonoids and phenolics improve gut health, feed efficiency, and disease resistance (Kour et al., 2024).

#### 3.3.1 *Azolla* in dairy and meat production

*Azolla* supplementation in dairy cattle diets improves milk yield, enhances milk quality, and reduces feed costs. Replacing 15–25% of commercial feed with *Azolla* in crossbred cows increased milk and fat percentage and yield by 7–13%, while reducing feed costs by 20–25% (Katole et al., 2017; Bujak and Bujak, 2022; Nasir et al., 2022; Alebachew Chekol et al., 2024). A 10–15% replacement of conventional cattle feed increased milk yield by 15–20%, with improved fat and protein content (Herath et al., 2023). Fresh supplementation of up to 1 kg/day increased yield by 7–13%, with extended feeding (28–63 days) further enhancing production (Roy et al., 2018). In buffaloes, daily feeding of 1.5 kg *Azolla* increased milk yield by 15–20% (Meena et al., 2017), while supplementation with cottonseed cake raised output from 8.0 to 9.3 L/day (Chatterjee et al., 2013).

In beef cattle and meat production, feeding *Azolla* to cattle and goats for two months increased milk production by 10–15% and meat yield by 8–10% (Alebachew Chekol et al., 2024). Feeding 5% dried *Azolla* improved feed conversion efficiency by 20% and daily gain by ~16% in heifers (Roy et al., 2016). In Sahiwal calves (*Bos indicus*), substituting 15–30% of concentrate protein with *Azolla pinnata* significantly enhanced growth, particularly in winter (Bhatt et al., 2021). The substitution of groundnut cake nitrogen with *Azolla* in buffalo calves improved daily weight gain, while 25% protein replacement in Murrah bulls’ concentrate had no negative effect.

Studies on small ruminants confirm that *Azolla* can partially replace protein sources in their diets. In Black Bengal goat, replacing 50% of concentrate with sun-dried *Azolla* caused severe diarrhea, but up to 20% inclusion was tolerated without adverse effects (Tamang and Samanta, 1995). In Jalauni lambs, *Azolla* replaced 25% of mustard cake protein without impacting nutrient digestibility (Das et al., 2017). Similarly, in Mecheri lambs, 10% *Azolla* in concentrate feed had no effect on dry matter intake, average daily gain, or feed efficiency (Sankar et al., 2020). For Corriedale sheep, diets replacing 25% of linseed cake with 6% *Azolla* showed no negative impact on performance (Ahmed et al., 2016). In goats, up to 15% sun-dried *Azolla* could be included in concentrate feed without adverse effects (Sajjan Sihag et al., 2018). Goats supplemented with 15% *Azolla* maintained digestible crude protein and nutrient intake (Sajjan Sihag et al., 2018; El Naggar and El-Mesery, 2022).

In pigs, *A. filiculoides* partially replaced soybean protein at 15–30%, leading to reduced growth in the early phase but improved compensatory growth during finishing (Becerra et al., 1990). Optimal *Azolla* replacement rates were 10% in the growing phase and 20% in the finishing phase, with higher inclusion levels negatively affecting weight gain and feed conversion efficiency (Durán, 1994). *Azolla pinnata* inclusion up to 20% in pig diets reduced feed costs while maintaining weight gain (Cherryl et al., 2013).

In other monogastric animals, the beneficial effects of *Azolla* have been studied in horses and rabbits. In Marwari stallions, replacing 10% of concentrate protein with *A. pinnata* had no effect on body weight or nutrient digestibility, supporting its suitability as a protein supplement (Songara et al., 2018). Similarly, supplementing rabbit feed with 1.5–3% *A. pinnata* in place of wheat bran and lucerne meal maintained normal growth performance (Sireesha et al., 2017).

#### 3.3.2 *Azolla* as poultry feed

*Azolla* is a sustainable poultry feed rich in protein, essential amino acids, vitamins, and bioactive compounds, enhancing growth performance and feed efficiency. A 5% *Azolla* inclusion enhanced broiler weight gain and feed efficiency (Parthasarathy et al., 2001), while 10% increased weight gain and reduced feed intake (Alalade and Iyayi, 2006). A 7.5% inclusion improved body weight by 2.6% (Prabina and Kumar, 2010), with optimal growth at 5–10%. *Azolla* supports digestion and gut microbiota in poultry, enhancing digestibility at 10–15% inclusion (Samad et al., 2020). Broilers fed 10% *Azolla* gained 1810 g versus 1270 g on conventional feed (Rai et al., 2012), with improved digestibility linked to increased duodenal thickness (Rana et al., 2017). A 5–7% *Azolla* diet with multivitamins and acidifiers lowered feed conversion ratio, mortality, and costs while boosting profit (Bolka, 2011; Islam and Nishibori, 2016). Previous studies reported that the *Azolla* fiber was more digestible than rice bran (Joysowal et al., 2018) and supported metabolism, immunity, and gut health in chickens and safety was confirmed up to 7% inclusion (Mishra et al., 2016). Broilers fed 10% *Azolla* showed higher Newcastle Disease antibody titers (Prabina and Kumar, 2010), while a 5.5% diet enhanced immune markers in turkeys (Bhattacharyya et al., 2016). *Azolla* supplementation (5–10%) boosted immunity, likely due to its carotenoids, minerals, and nitrogen-fixing *Anabaena* (Chichilichi et al., 2015; Mishra et al., 2016).

Previous studies confirmed that *Azolla* enhances egg production and quality without adverse effects up to 20% inclusion (Rathod et al., 2013; Chisembe et al., 2020; Alagawany et al., 2024). Layers fed 100 g/day produced more eggs at lower costs (Kannaiyan and Kumar, 2005). Ducks on a 10–20% *Azolla* diet showed increased egg weight and better feed conversion (Swain et al., 2022). A 5% inclusion improved egg production (53.2 vs. 49.9 on concentrate, 47 on forage) and body weight (Alebachew Chekol et al., 2024). Fresh *Azolla* supported growth in backyard poultry, while its carotenoids enhanced yolk pigmentation and egg yield (Ali and Leeson, 1995). Additionally, *Azolla* enhances meat quality and overall health outcomes in poultry. For example, a 5% *Azolla* diet significantly increased dressing percentage (Basak et al., 2002), while a 4.5% diet improved giblet yield and reduced serum cholesterol (Balaji et al., 2009). Broilers fed 5–10% *Azolla* exhibited better meat color and reduced cooking loss (Abdelatty et al., 2020). However, excessive supplementation may cause a greenish tint in meat.

#### 3.3.3 *Azolla* in aquaculture and fish farming

*Azolla* has been extensively investigated as a potential feed supplement in aquaculture due to its high protein content, balanced amino acid profile, and natural pigments, which contribute to improved growth rates and enhanced coloration in fish species such as tilapia and carp (Marzouk et al., 2023) (**Table 2**). Additionally, the incorporation of *Azolla* in fish diets has been shown to enhance water quality by absorbing excess nutrients and mitigating algal blooms, thereby creating a more balanced aquatic environment (Kollah et al., 2016). Various freshwater fish species, including tilapia (*Oreochromis niloticus*), redbelly tilapia (*Coptodon zillii*), catfish, fringed-lipped carp (*Labeo fimbriatus*), calbasu (*Labeo calbasu*), and Thai silver barb, have been successfully fed *Azolla*-based diets in controlled experimental settings (Das et al., 2018; Yohana et al., 2023). Examples of previous studies assessing the impact of *Azolla* supplementation on fish growth and survival are provided in **Table 2**.Previous studies reported that Tilapia can tolerate up to 20% *Azolla* in their diet without growth impairment (Magouz et al., 2020; Alebachew Chekol et al., 2024), with a recommended daily intake of 100 g for juveniles and 200 g for adults (El-Sayed and Garling, 1988). Inclusion levels vary by species, with rohu tolerating up to 50%, Thai silver barb 25%, fringed-lipped carp 40%, and calbasu 30%. A 25% *A. pinnata* diet in Thai silver barb showed no significant differences in growth or survival compared to controls (Das et al., 2018; Yohana et al., 2023).

**Table 2:** Examples of previous studies showing the impact of *Azolla* diet on fish.

*Azolla* species	Fish	Method	Findings	Citation
*A. microphylla*	*Oreochromis niloticus*	Tilapia were fed diets with varying levels of *Azolla* meal for 90 days.	Fish growth declined when *Azolla* meal exceeded 20% in diet. However, fatty acid content improved.	(Abou et al., 2013)
*A. pinnata*	*Tilapia zillii*	Fish of different sizes were fed fresh and dried *Azolla* meal for 8 weeks.	Fish growth declined when *Azolla* meal exceeded 25% in diet.	(Abdel‐Tawwab, 2008)
*A. filiculoides*	*Labeo rohita*	Examined six *Azolla* species’ growth potential and their efficacy as a feed ingredient in a 150-day trial.	*Azolla* mixture at 25% inclusion showed highest fish weight gain and best specific growth rate.	(Datta, 2011)
*A. pinnata*	*Oreochromis niloticus*	Fresh green *Azolla* replaced 0-40% of commercial feed in Nile tilapia diets for 70 days.	20% replacement resulted in the best growth, enzyme activity, and protein efficiency ratio.	(Refaey et al., 2023)
*A. filiculoides*	*Tilapia nilotica*	Tilapia were fed diets containing different proportions of *Azolla* over 3 weeks.	Fish growth was reduced at higher *Azolla* inclusion levels, but fatty acid composition improved.	(Shiomi and Kitoh, 2001)
*A. filiculoides*	*Oreochromis niloticus*	Nutritional composition and fatty acid profile of tilapia fed *Azolla* diets were analyzed over 90 days.	High *Azolla* inclusion led to reduced growth but improved omega-3 fatty acid composition.	(Abou et al., 2010)
*A. pinnata*	*Oreochromis niloticus*	Fish meal was substituted with *Azolla* at varying levels for fingerling and adult tilapia.	*Azolla* inclusion reduced fish growth and feed utilization, with body protein and lipid content negatively correlated to its level in the diet.	(El‐Sayed, 1992)
*A. filiculoides*	*Oreochromis niloticus*	Tilapia were fed diets containing 0-50% *Azolla* meal over 90 days in earthen ponds.	Growth reduced above 20% *Azolla*, but fatty acid profile improved	(Youssouf et al., 2011)
*A. pinnata*	Various finfish species	Reviewed literature on *Azolla* meal inclusion in finfish diets (tilapia, catfish, cyprinids).	*Azolla* meal inclusion of 10-45% had positive effects, but species-specific responses varied.	(Mosha, 2018)
*A. filiculoides*	Oreochromis niloticus	Tilapia were fed diets with different levels of *Azolla* meal in earthen ponds.	Higher *Azolla* inclusion reduced growth but improved fish fatty acid profile and reduced	(Youssouf et al., 2012)
*A. microphylla*	*Oreochromis niloticus*	Compared fish growth with diets containing 15-45% *Azolla* meal in a recirculating system.	*Azolla* addition up to 45% in the diet supported growth and it was least expensive diet among tested diets	(Fiogbé et al., 2004)
*A. pinnata*	*Nile tilapia*	Investigated fish performance on *Azolla*-based diets with fishmeal substitution.	Optimal *Azolla* inclusion improved fish health but excessive levels hindered growth.	(Leonard et al., 1998)
*A. pinnata*	*Nile tilapia*	Fish was fed on fresh and dried *Azolla* as partial or full replacement of diet	Fish fed only fresh *Azolla* showed poor growth with reduced lipid and protein content, but a 50% inclusion in the control diet maintained normal growth	(Tharwat, 1999)
*A. africana*	*Oreochromis niloticus*	Sun-dried *Azolla* africana was incorporated into practical diets for Nile tilapia fingerlings.	Growth was improved up to 20% inclusion but declined at higher levels.	(Fasakin et al., 2001)
*A. filiculoides*	*Oreochromis niloticus*	Nile tilapia were reared under varying *Azolla* cover (0-90%) in earthen ponds for 90 days.	Fish survival remained high, and indirect effects of *Azolla* on phytoplankton and zooplankton influenced growth.	(Abou et al., 2012)
*A. pinnata*	Catfish	*Azolla* was grown in catfish wastewater to assess nutritional value and phytoremediation potential.	*Azolla* exhibited high growth and nutrient absorption, reducing Total Nitrogen and Total Phosphate levels.	(Said et al., 2012)
*A. pinnata*	*Labeo fimbriatus*	*Azolla* was incorporated at 10-40% in fish diets over 75 days.	Up to 40% *Azolla* inclusion reduced feed costs without affecting fish growth or survival.	(Gangadhar et al., 2015)
*Azolla* spp	Various fish species	Reviewed *Azolla*’s role in aquaculture, focusing on its protein content and feed potential.	*Azolla* is a rich protein source but has digestibility issues for some fish species.	(Yohana et al., 2023)
*A. pinnata*	*Oreochromis niloticus*	Fermented *Azolla* was used in tilapia fry diets at varying inclusion levels.	20% fermented *Azolla* inclusion showed the best growth and feed efficiency.	(Hundare et al., 2018)
*Azolla* spp	*Tilapia nilotica*	Investigated *Azolla*’s impact on aquaculture sustainability and economic feasibility.	Positive growth effects were observed in fish, showing potential as a protein source.	(Shernazarov et al., 2024)
*A. filiculoides*	*Oreochromis niloticus*	Nile tilapia were stocked at different densities and fed *Azolla* diets.	Nile tilapia could be raised at a density of 3 fish/m^2^ with 30-40% *Azolla* inclusion to improve production	(Abou et al., 2007)

Moderate *Azolla* inclusion enhances feed conversion ratio, protein efficiency, and energy utilization, while excessive levels may impair digestion due to antinutritional factors like phytates and fibers (Yohana et al., 2023). Its bioactive compounds, including phenols and flavonoids, support antioxidant and immunostimulatory functions (Lumsangkul et al., 2022). *Azolla* supplementation also boosts goblet cell production, strengthening the mucosal barrier and enhancing disease resistance in fish (Lumsangkul et al., 2022). In biofloc systems, Nile tilapia fed 100 g/kg *Azolla* exhibited improved immune responses and growth performance. While *Azolla* is promising as an aquaculture feed, but its amino acid balance still needs improvement, and the negative effects of its anti-nutritional compounds need to be reduced (Lumsangkul et al., 2022; Yohana et al., 2023).

## 4 *Azolla*’s contribution to climate resilience

### 4.1 CO_2_ absorption potential

*Azolla* is an efficient natural sink for atmospheric CO_2_ due to its rapid growth, high biomass accumulation, and symbiosis with *A. azollae*, which enables continuous nitrogen fixation without external inputs *(* (Vroom et al., 2024) (Kour et al., 2024). Its integration into wetlands and rice paddies enhances carbon cycling, soil carbon storage, and long-term ecosystem stability (Sadeghi et al., 2014).

Evidence shows that *Azolla* can sequester CO_2_ at rates comparable to, or greater than, terrestrial plants. The Eocene “*Azolla* bloom” contributed significantly to global cooling, highlighting its historic efficiency as a CO_2_ sink (Yuan et al., 2024). Modern studies estimate that a 1-ha *Azolla* Pond captures 21,266 kg CO_2_ annually, and that 1,018,023 km² (one-fifth the Amazon) could offset current global CO_2_ increases. Compared to terrestrial ecosystems, which absorb 20–30% of anthropogenic emissions, *Azolla* ponds remove CO_2_ 18 times more efficiently than an equivalent Amazon forest area (Hamdan and Houri, 2022).

Practical applications extend beyond sequestration. As a biofertilizer, *Azolla* improves soil quality and reduces the need for inorganic fertilizers that contribute to GHG emissions (Hamdan and Houri, 2022). In poultry farming, replacing 50% of feed with *Azolla* reduced CO_2_ by 35%, N_2_O by 22.3%, and CH_4_ by 4.7%, corresponding to a 28.5% reduction in global warming potential per 1,000 birds (Espino and Bellotindos, 2020). Similarly, cultivation trials reported annual fixation of 1.86 t CO_2_ and 0.33 t N ha^-^¹, providing dual climate and agronomic benefits (Brinkhuis and Bijl, 2014). In Sri Lanka, expanded *Azolla* use in paddy fields could mitigate 509,422 t of CO_2_ annually (Surenthiran and Loganathan, 2012), while *A. filiculoides* sequesters 32.54 metric tons CO_2_ ha^-^¹ year^-^¹, surpassing grassland, forest, and algae (Dawson and Smith, 2007).

CO_2_ enrichment experiments (380–680 ppm) further demonstrated enhanced *Azolla* biomass, confirming its scalability as a mitigation strategy (Cheng et al., 2010). During the *Azolla* interval, mean sea surface temperatures dropped from 13°C to 10°C, demonstrating its historical role in climate regulation (Brinkhuis et al., 2006). Sensitivity analyses indicate that optimal *Azolla* cultivation could require sequestration areas between 763,518 and 1,527,036 km² to significantly counteract atmospheric CO_2_ rise. Given its historical impact on climate stabilization and its efficiency in CO_2_ capture, *Azolla*-based strategies could contribute significantly to mitigating global warming and enhancing carbon management in agroecosystems (Hamdan and Houri, 2022).

### 4.2 Impact on methane emissions in rice cultivation

Agriculture is a major contributor to greenhouse gas (GHG) emissions, particularly CO_2_, CH_4_, and N_2_O (Chataut et al., 2023). CO_2_ results from microbial decay and organic matter oxidation, while CH_4_, a potent GHG with 20–60 times the global warming potential of CO_2_, is produced under anaerobic conditions in flooded rice paddies, livestock digestion, and manure storage (Smith et al., 2008). N_2_O arises from nitrogen transformations in soil, particularly under excessive fertilization (Xiao et al., 2024). Rice paddies contribute ~20% of global CH_4_ emissions, necessitating mitigation strategies. *Azolla* offers a natural means of reducing CH_4_ emissions in rice systems. By releasing oxygen, absorbing excess nutrients, and altering soil redox potential, *Azolla* suppresses methanogenesis while maintaining or improving rice yields (Razavipour et al., 2018). Field studies consistently report 30–60% reductions in CH_4_ emissions when *Azolla* is used as green manure or a floating cover (Serag et al., 2000a). In a three-year double rice cropping study, *Azolla* integration lowered CH_4_ emissions, reduced nitrogen fertilizer requirements, and maintained yields, largely due to improved soil oxygenation (Xu et al., 2017).

Synergistic practices further enhance these benefits. In Japan, combining *Azolla* with poultry-litter biochar increased rice yields by 27–75% while cutting CH_4_ by ~25% and N_2_O by up to 98% (Kimani et al., 2020). In India, dual cropping with *Azolla* reduced CH_4_ flux by 40% compared to urea fertilization alone, confirming the role of oxygen release in lowering emissions (Bharati et al., 2000). Laboratory studies also show that soils treated with *Azolla* and urea exhibit higher CH_4_ oxidation than urea alone, due to oxygen supplied by cyanobacteria (Adhya et al., 2000; Sood et al., 2012). Comparisons with other organic amendments reveal that CH_4_ efflux per grain yield is lowest in *Azolla+*urea systems, outperforming Sesbania, farmyard manure, and urea alone (Adhya et al., 2000). Although some Chinese studies reported higher CH_4_ emissions under *Azolla* dual cropping (Ying et al., 2000), soil type and nutrient status strongly influence outcomes. For example, Indian soils tend to produce lower CH_4_ flux under similar management (Gollany et al., 2015). Alternative integrated models, such as rice–fish culture, also reduce CH_4_ by improving soil aeration, lowering emissions by ~35% compared to conventional paddies (Kollah et al., 2016). Combining *Azolla* with such climate-smart practices could provide scalable, site-specific solutions for mitigating CH_4_ emissions while enhancing rice productivity and sustainability.

### 4.3 Bioremediation and pollution control

*Azolla* is an efficient phytoremediator with the ability to absorb and accumulate heavy metals from contaminated water. Both living and dead biomass are effective in removing pollutants, owing to mechanisms that include passive adsorption onto cell walls and active metabolic uptake (Sood et al., 2012). Its rapid growth and high bioaccumulation potential enable the uptake of Pb, Cd, As, Cr, and Hg from industrial and agricultural effluents, with removal efficiencies reported up to 80% (Vroom et al., 2024). The mechanism of heavy metal uptake involves passive adsorption onto cell walls as well as active metabolic absorption, making *Azolla* a suitable candidate for the remediation of polluted wetlands, rivers, and agricultural runoff areas (Sadeghi et al., 2014).

Field studies show that *A. pinnata* removes 70–94% of heavy metals from effluents, with tissue concentrations up to 740 mg/kg (Rai, 2008). *Azolla filiculoides* efficiently absorbs Cr, Pb, Zn, Hg, Cu, Cd, Ag, and Ti from wetland environments, demonstrating its potential for metal removal in natural water bodies (Hassanzadeh et al., 2021). Hydroponic studies indicate species-specific differences: *A. caroliniana* accumulated up to 284 mg/kg of As, while *A. filiculoides* accumulated only 54 mg/kg (Zhang et al., 2008). Another study reported that *A. caroliniana* also bioaccumulates Hg and Cr (III and VI), with tissue concentrations between 71 and 964 mg/kg dry weight (Bennicelli et al., 2004).

The tolerance of *Azolla* to heavy metals varies among species. For example, *A. filiculoides* demonstrated the highest tolerance to Cr exposure, retaining 72% of its control biomass under contamination (Arora et al., 2006). Nonetheless, heavy metals reduce growth, chlorophyll, and protein content, with Cd and Pb showing the greatest toxicity (Sarkar and Jana, 1986; Guo-Xin et al., 2003; Sood et al., 2012). Stress responses include reduced photosynthesis, O_2_ evolution, and enzyme activity, while detoxification is supported by increased phenolics and PAL activity (Dai et al., 2006). Similarly, Hg toxicity in *A. pinnata* reduces chlorophyll a, protein, RNA, DNA, and nutrient uptake, further compromising growth and metabolic functions (Rai and Tripathi, 2009). Copper particularly affects photosystem II efficiency (Sanchez-Viveros et al., 2010).

Heavy metal exposure also induces ultrastructural damage in *Azolla*, affecting organelles at the cellular level. Structural disruptions include chloroplast swelling, mitochondrial deformation, chromatin condensation, and nuclear membrane disintegration (Sela et al., 1988, 1990; Sood et al., 2012). Copper accumulates preferentially in roots, whereas Cd is evenly distributed in plant tissues, forming detoxification aggregates with PO_4_ and Ca (Sela et al., 1988). Cadmium localizes in the epidermis, cortex, and bundle cell walls within 77 hours, leading to the formation of electron-dense granules (Sela et al., 1990). Lead precipitation in *A. filiculoides* was primarily localized in vacuoles, with higher accumulation in mature leaves (Benaroya et al., 2004). In *A. pinnata*, Pb exposure caused frond compactness, stomatal closure, and epicuticular wax deposition, though these effects were mitigated by Fe supplementation (Gaumat et al., 2008).

Beyond metal accumulation, *Azolla* has demonstrated potential for biosorption. Studies show that dead or pretreated *Azolla* biomass effectively removes Cs, Sr, Pb, Zn, Ni, Cu, Au, Cd, and Cr from contaminated water sources (Sood et al., 2012). The bioaccumulation capacity of *Azolla* is influenced by metal concentration and environmental conditions (Dai et al., 2006; Sood et al., 2012). Hydroponic experiments have shown that *A. filiculoides* can remove Cr^6+^ with a maximum adsorption capacity of 20.2 mg/g at pH 2 and 32°C, while Ni uptake reached 27.9 mg/g at 60% saturation (Zhao and Duncan, 1998). Pb removal efficiency by *A. filiculoides* remained at approximately 90% between 10 and 50°C, with minimal influence from biomass concentration (Sanyahumbi et al., 1998). Furthermore, *A. filiculoides* has demonstrated a 99.9% efficiency in gold biosorption at pH 2 (Sood et al., 2012).

Apart from heavy metal removal, *Azolla* effectively removes excess nutrients from wastewater, reducing eutrophication risks. *Azolla filiculoides* has been reported to extract up to 122 kg of phosphorus per hectare annually (Vroom et al., 2024). Sequential treatment using *Landoltia punctata* followed by *A. filiculoides* achieved complete NH_4_ and NO_3_ removal and a 93% reduction in PO_4_, significantly lowering wastewater toxicity (Miranda et al., 2020). In addition, *Azolla* plays a crucial role in domestic wastewater treatment by removing nitrogen and phosphorus, thereby improving water quality for irrigation (Muradov et al., 2014). Laboratory studies further indicate that *A. filiculoides* effectively removes textile dyes such as Congo Red, Acid Red 88, Acid Green 3, Acid Orange 7, and Basic Orange from industrial effluents (Tan et al., 2010; Sood et al., 2012). The biosorption potential of *Azolla* is enhanced by its rapid growth and high surface area, making it an eco-friendly alternative to conventional wastewater treatment methods (Sood et al., 2012).

## 5 Other uses of *Azolla*

*Azolla* is a promising and sustainable biofuel feedstock due to its rapid growth, high lipid content, and adaptability to various conversion processes (Arora et al., 2022; Ramesh and Rajendran, 2022). Pyrolysis of *Azolla* yields hydrocarbons, including straight-chain alkanes, making it a potential diesel substitute. However, its high moisture content requires drying, and heavy metal emissions must be managed. Activated carbon catalysts improve both bio-oil yield and quality.

Transesterification is another widely studied method for biodiesel production. Crude oil extracted from dried *Azolla* biomass can be processed through acid transesterification to produce fatty acid methyl esters with properties like conventional diesel. Efficiency depends on maintaining optimal reaction temperatures (47–60°C) and reactant ratios, which require further study (Arora et al., 2022; Prabakaran et al., 2022). Hydrothermal liquefaction and torrefaction convert *Azolla* into bio-crude oil under high temperature and pressure. Torrefaction reduces moisture content and enhances fuel stability. Ethanol production involves hydrolysis, yeast isolation, and fermentation: acid treatment breaks down the biomass, sugars are released, and microbial fermentation produces ethanol. Additionally, microbial fuel cells using *Azolla* biomass and pyrolyzed biochar as anodes have shown potential for bioelectricity generation while reducing chemical oxygen demand (Miranda et al., 2016; Arora et al., 2022; Prabakaran et al., 2022).

*Azolla* is also a promising feedstock for bioplastics due to its rapid growth, high biomass yield, and diverse biochemical composition (Kouchakinejad et al., 2024). Unlike corn and sugarcane, *Azolla* does not compete with food production, making it a more sustainable alternative. Its cellulose and hemicellulose support bioplastic synthesis through chemical and enzymatic hydrolysis, while protein and lipid fractions can produce protein-based and lipid-derived bioplastics, including polyhydroxyalkanoates (PHA).

Blending *Azolla* biomass with poly (lactic acid) or starch can improve biodegradability and mechanical performance. Microbial fermentation also enables PHA production, providing a renewable alternative to petroleum-based plastics. A biorefinery approach, integrating bioplastic production with biofuels and biofertilizers, maximizes resource efficiency. Given its rapid biomass doubling and adaptability to pond and bioreactor cultivation, *Azolla* offers a scalable and efficient pathway for sustainable bioplastic production (Supriya et al., 2023; Kouchakinejad et al., 2024).

## 6 Challenges and limitations in *Azolla* utilization

Despite its numerous benefits, large-scale cultivation of *Azolla* faces several constraints related to environmental requirements, ecological risks, preservation, and economic feasibility.

Environmental and agronomic constraints: *Azolla* thrives under specific conditions—temperatures of 20–30°C, high humidity, adequate sunlight, and still or slow-moving water (Watanabe et al., 1989). Fluctuations in climate, seasonal variations, and poor water quality reduce productivity in open systems (Sadeghi et al., 2013). Continuous nutrient uptake can deplete nitrogen and phosphorus, requiring supplementation to sustain biomass yields (Razavipour et al., 2018). Growth is also inhibited under highly acidic or alkaline pH (Madeira et al., 2013). Competition from algae and aquatic weeds, coupled with risks of stagnation, oxygen depletion, and biomass decay, further limit performance (Vroom et al., 2024).

Ecological risks and invasiveness: Certain species, such as *A. filiculoides* and *A. pinnata*, can proliferate rapidly, doubling biomass every 3–5 days under optimal conditions (Vroom et al., 2024). Unchecked growth forms dense mats that block light, lower dissolved oxygen, and disrupt aquatic ecosystems (Sadeghi et al., 2014). Invasive infestations in wetlands, irrigation canals, and lakes have displaced native vegetation and altered water chemistry (Madeira et al., 2013). Mitigation requires controlled cultivation, floating containment systems, routine harvesting, and ecological risk assessments before introduction into new environments.

Preservation and shelf-life limitations: Fresh *Azolla* decomposes within days, making storage and transport difficult for feed and biofertilizer use (Razavipour et al., 2018). Various preservation techniques, including sun-drying, freeze-drying, and ensiling, have been explored; however, these methods often result in nutrient degradation and reduced digestibility for livestock consumption (Sadeghi et al., 2014). Long-term storage demands airtight facilities to prevent fungal contamination. Cost-effective preservation strategies—such as optimized dehydration or fermentation-based methods—are still under development.

Safety and consistency concerns: *Azolla*’s ability to accumulate heavy metals, while valuable for phytoremediation, poses risks when biomass from polluted environments is used for feed or fertilizer (Madeira et al., 2013) Furthermore, nutrient composition varies with species, climate, and soil conditions, making it difficult to ensure consistent feed quality. Standardized cultivation protocols are required to deliver predictable nutritional value (Vroom et al., 2024).

Logistical and regulatory barriers: High water content makes transport of fresh biomass costly and inefficient, requiring drying or processing facilities for distribution. In some regions, *Azolla* is classified as an invasive species, restricting its cultivation and use (Razavipour et al., 2018). Clear regulatory frameworks and risk assessments are therefore essential to balance utilization with ecological safeguards.

Adoption and economic limitations: Despite proven agronomic benefits, adoption among farmers remains low due to limited awareness, technical expertise, and uncertainties about labor requirements (Watanabe et al., 1989). Initial investment in ponds, harvesting, and processing infrastructure also discourages small-scale farmers, even though long-term savings on fertilizers and feed are possible (Razavipour et al., 2018). High water content, short shelf life, and the need for preservation or processing facilities further increase production and transport costs, limiting economic feasibility. To overcome these barriers, farmer training, cost-sharing schemes, and targeted incentive programs—such as subsidies, integration into carbon credit markets, and inclusion in climate-smart agriculture policies—are needed to promote wider adoption and improve sustainability.”.

## 7 Future prospects and research directions

*Azolla* is a promising resource for sustainable agriculture, but realizing its large-scale potential requires advances in genetics, cultivation technologies, and commercial integration.

Genetic and biotechnological improvement: Selective breeding, molecular breeding, and gene editing hold potential to enhance biomass yield, nitrogen fixation efficiency, and tolerance to abiotic stresses such as drought, salinity, and temperature fluctuations (Madeira et al., 2013; Vroom et al., 2024). Future work should also explore microbial symbiosis optimization and metabolic engineering to develop high-yielding, stress-resilient strains with consistent nutrient composition for diverse environments.

Precision agriculture and digital tools: Integrating *Azolla* cultivation with remote sensing, drone-based monitoring, and modelling based analytics could improve nutrient management, predict biomass productivity, and optimize harvesting schedules. Automated water-quality sensors and modeling can further minimize risks of uncontrolled growth and invasiveness, enabling more efficient and scalable production systems.

Commercial applications and sustainability pathways: Beyond biofertilizers, *Azolla* offers opportunities in livestock feed, aquaculture, wastewater treatment, and carbon markets. Its high protein content supports poultry, cattle, swine, and fish diets, reducing dependence on conventional feeds. As a biofertilizer, it aligns with organic and regenerative farming systems, while its CO_2_ sequestration capacity creates potential for participation in carbon credit schemes. In parallel, its ability to absorb heavy metals makes it a valuable tool in industrial wastewater treatment and pollution control.

## 8 Conclusion

*Azolla* is a multifunctional resource that supports sustainable agriculture and climate resilience. Its symbiosis with *A. azollae* enables efficient nitrogen fixation, reducing dependence on synthetic fertilizers while enhancing soil health, conserving water, and suppressing weeds and pests. As a high-protein feed, *Azolla* improves livestock, poultry, and aquaculture performance, though anti-nutritional factors and compositional variability remain challenges. Beyond agriculture, *Azolla* contributes to climate change mitigation through rapid carbon sequestration and reduced methane emissions in rice systems, while also offering bioremediation potential for polluted waters. Its emerging applications as a feedstock for biofuels and bioplastics further highlight its industrial value. However, large-scale use faces barriers such as short shelf life, high water content, and ecological risks from invasiveness.

Overall, *Azolla* represents a low-cost, eco-friendly tool with wide-ranging benefits across food production, climate mitigation, and environmental management. Addressing preservation, standardization, and ecological safeguards, along with advances in genetics and precision cultivation, will be critical to unlocking its full potential as a cornerstone of climate-smart agriculture.
